# Coupling integrin dynamics to cellular adhesion behaviors

**DOI:** 10.1242/bio.036806

**Published:** 2018-08-15

**Authors:** Catherine G. Galbraith, Michael W. Davidson, James A. Galbraith

**Affiliations:** 1 Oregon Center for Spatial Systems Biomedicine, Department of Biomedical Engineering, Oregon Health Science University, Portland, OR 97201, USA; 2 National High Magnet Laboratory, Florida State University, Tallahassee, FL 32310, USA

**Keywords:** Integrins, Single molecule, Super-resolution microscopy, Cell adhesion

## Abstract

Visualizing fluorescent proteins is essential for understanding cellular function. While advances in microscopy can now resolve individual molecules, determining whether the labeled molecules report native behaviors and how the measured behaviors can be coupled to cellular outputs remains challenging. Here, we used integrin alpha-beta heterodimers – which connect extracellular matrix (ECM) and the cytoskeleton – to quantify the mobility and conformation of labeled integrins. We found that while unlabeled and labeled integrins all localized to adhesions and support anchorage-dependent cell function, integrin mobility decreased when the beta rather than the alpha subunit was labeled. In contrast to unlabeled and alpha labeled subunits, beta labeled subunits changed cellular behavior; decreasing protrusive activity and increasing adhesion size and the extent of cell spreading. Labeling the beta subunit changed the integrin conformation, extending the molecule and exposing an epitope that is revealed by activation with Mn^2+^ treatment. Our findings indicate labeling induced changes in dynamic integrin behavior alter molecular conformation as well as cellular adhesion-dependent function to demonstrate a coupling between molecular inputs and distinct cellular outputs.

This article has an associated First Person interview with the first author of the paper.

## Abstract (precis)

**Summary:** Our findings indicate labeling induced changes in dynamic integrin behaviors alter molecular conformation and cellular adhesion-dependent function to demonstrate a coupling between molecular inputs and distinct cellular outputs.

## INTRODUCTION

Integrins are bi-directional signaling molecules that form attachments between the extracellular matrix (ECM) and the cytoskeleton. They are alpha-beta heterodimers, with a large extracellular globular head containing a ligand-binding domain formed by noncovalent interactions between the alpha and beta subunits. The head sits atop two multi-domain legs, each composed of a single spanning transmembrane domain and a short cytoplasmic tail. Crystal structures have shown that integrins adopt three major conformational states (Askari et al., 2009). In the first state, the molecule is bent with the ligand-binding domain close to the legs; it is an inactive conformation with a low affinity for ligand. In the second state, the molecule is extended into a primed conformation with a high affinity for ligand. The molecule is also extended in the third state, but in this conformation the headpiece is open and ligand bound (Askari et al., 2009; Carman and Springer, 2003; Moore et al., 2018; Takagi et al., 2002a,b). Cells spatially and temporally manipulate the transition of integrins between these conformations to regulate anchorage-dependent events such as adhesion, spreading and migration. Integrin conformation can also be experimentally manipulated by treatment with Mn^2+^, but Mn^2+^ results in a mixture of open and closed headpieces, suggesting that it primes the molecule and elevates affinity but does not activate as completely as ligand binding (Carman and Springer, 2003).

Extensive studies have used GFP labeled integrins and Mn^2+^ to study cell anchorage-dependent function (Ballestrem et al., 2001; Parsons et al., 2008; Plançon et al., 2001; Rossier et al., 2012). Independently of whether the alpha or the beta subunit is labeled, labeled integrins have been shown to express on the cell surface and be capable of concentrating at adhesion complexes where they bind ECM and establish connections with the cytoskeleton (Laukaitis et al., 2001; Tsuruta et al., 2002). These adhesion complexes increase in size in response to Mn^2+^ treatment (Ballestrem et al., 2001), and together the data suggest that labeled integrins function as expected. However, these studies typically only report the ensemble behaviors of populations of labeled integrins (Brown et al., 2006; Chen et al., 2012; Wehrle-Haller, 2007); the behaviors of individual molecules are usually not reported unless they are spatially segregated into some type of adhesion complex (Rossier et al., 2012; Spiess et al., 2018; Wiseman et al., 2004). Here, we quantified the mobilities of individual molecules within large populations to investigate whether labeling perturbs molecular behaviors and how the behaviors of labeled proteins compare to unlabeled proteins in producing the cellular outputs of protrusion, spreading, and adhesion (Jaqaman et al., 2016). We identified connections between molecular mobility and cellular protrusive activity that are coupled to integrin conformation changes and increased cellular ligand affinity.

## RESULTS AND DISCUSSION

### Single-molecule mobility of integrins depends on which subunit is fluorescently labeled

Although labeled integrins localize to adhesion complexes, localization is a population level assessment of behavior (Ballestrem et al., 2001; Laukaitis et al., 2001) and we wanted to evaluate the behavior of labeled integrins at the single-molecule level. We transfected both alpha V and beta 3 integrins in CHOK1 cells, which do not endogenously express either subunit (Xu et al., 2011). While transfection of one subunit did not result in significant surface expression, simultaneous transfection of both subunits significantly enhanced expression, with the majority of the transfected subunits forming heterodimers with each other, suggesting that the level of endogenous subunits limited formation of new heterodimers (Rick Horwitz, personal communication). Only one of the subunits was labeled with the photoactivatible fluorophore mEos2, which was attached to the integrin cytoplasmic tails with optimized linkers (Jaqaman et al., 2016; Kanchanawong et al., 2010) and these plasmids are available from Addgene (see the Materials and Methods section for Addgene number and link to plasmid map). We stochastically photo-converted the labeled subunits and used super-resolution microscopy to localize the position of the integrins. Images from live cells were collected using multi-scale microscopy where single-molecule data was collected with 25 ms exposure at a frame rate of 40 Hz, and every 400th frame was toggled to conventional total internal reflection (TIRF) imaging of the cell surface to track protrusion (Jaqaman et al., 2016). Cells were selected for imaging based on having similar expression of the fluorescence intensity of an EGFP null vector that was co-transfected with the integrins. Importantly, post-imaging single-molecule quantification of expressed integrin demonstrated that this method of selection resulted in less variation in surface density of expressed integrin between cells than across the area of an individual cell (Jaqaman et al., 2016).

**Fig. 1.**
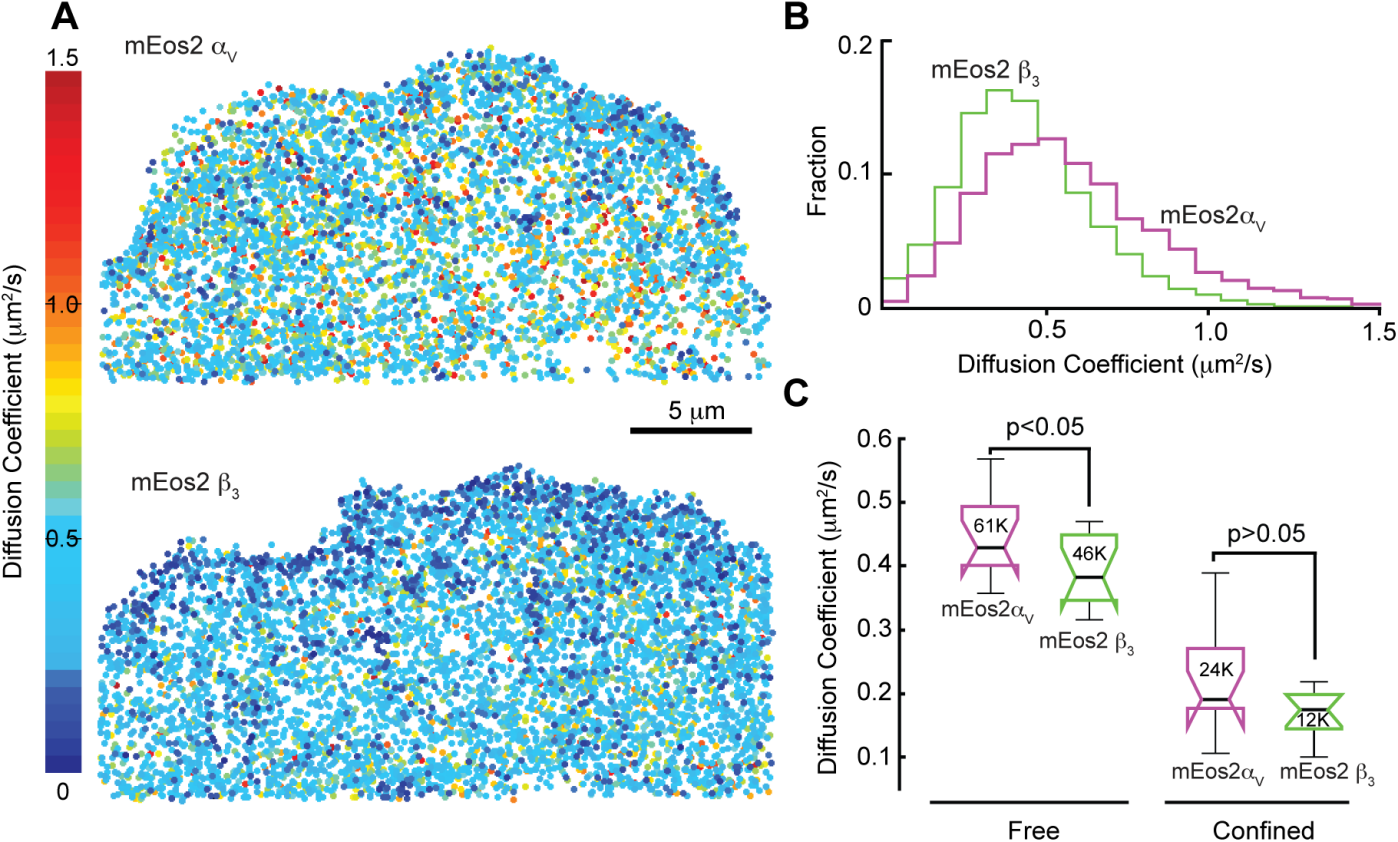
**Mobility of integrin molecules is slower when the beta subunit is fluorescently labeled.** (A) Cells were co-transfected with alpha V and beta 3 integrin subunits. Integrin molecules with labeled beta subunits have lower diffusion coefficients than integrins with labeled alpha subunits. Diffusion coefficients color-coded and plotted as points whose centroid indicate the mean position of the integrin during the lifetime of the fluorophore. (B) Distribution of diffusion coefficients from the 9237 molecules analyzed for the alpha label and 8874 molecules for the beta label in A. (C) Analysis of multiple cells show that free diffusion is significantly different between cells with alpha and beta labeled integrins but not for confined movement. *N*=11 cells each for alpha and beta labeled subunit conditions. Number inside each box and whisker is the number of molecules analyzed for each category.

We then measured the mobility of the labeled integrin by analyzing their trajectories using uTrack (Movie 1) (Jaqaman et al., 2008). For trajectories greater than 20 steps in length, we calculated diffusion coefficients and used moment spectrum scaling (MSS) to further classify mobility as confined, free, or directed diffusion (Fig. 1A) (Ewers et al., 2005; Ferrari et al., 2008; Jaqaman et al., 2008). We discovered that for all cells analyzed, the mobility of freely diffusing integrins was statistically lower when the expressed integrins had labeled beta subunits rather than labeled alpha subunits (Fig. 1A,B). Confined integrins had similar mobilities regardless of which subunit was labeled (Fig. 1C). The lower mobility of the diffusing beta labeled integrins was not reflective of a significant difference in integrin surface density between the labels. The average molecular density was 0.22±0.05 molecules/µm^2^ when the alpha subunit was labeled (*N*=11 cells) compared to 0.19±0.06 molecules/µm^2^ (*N*=11 cells) when the beta subunit was labeled, as measured by live-cell PALM (Jaqaman et al., 2016; Shroff et al., 2008). The surface density did not significantly change during the time course of the experiment, suggesting that the difference in mobility is not a result of different rates of integrin appearance or disappearance on the cell surface. Together these data indicate that labeling the beta subunit decreases the mobility of the integrins that are not already interacting with either a ligand or other proteins in adhesion complexes.

### Protrusive activity depends on which integrin subunit is fluorescently labeled

**Fig. 2.**
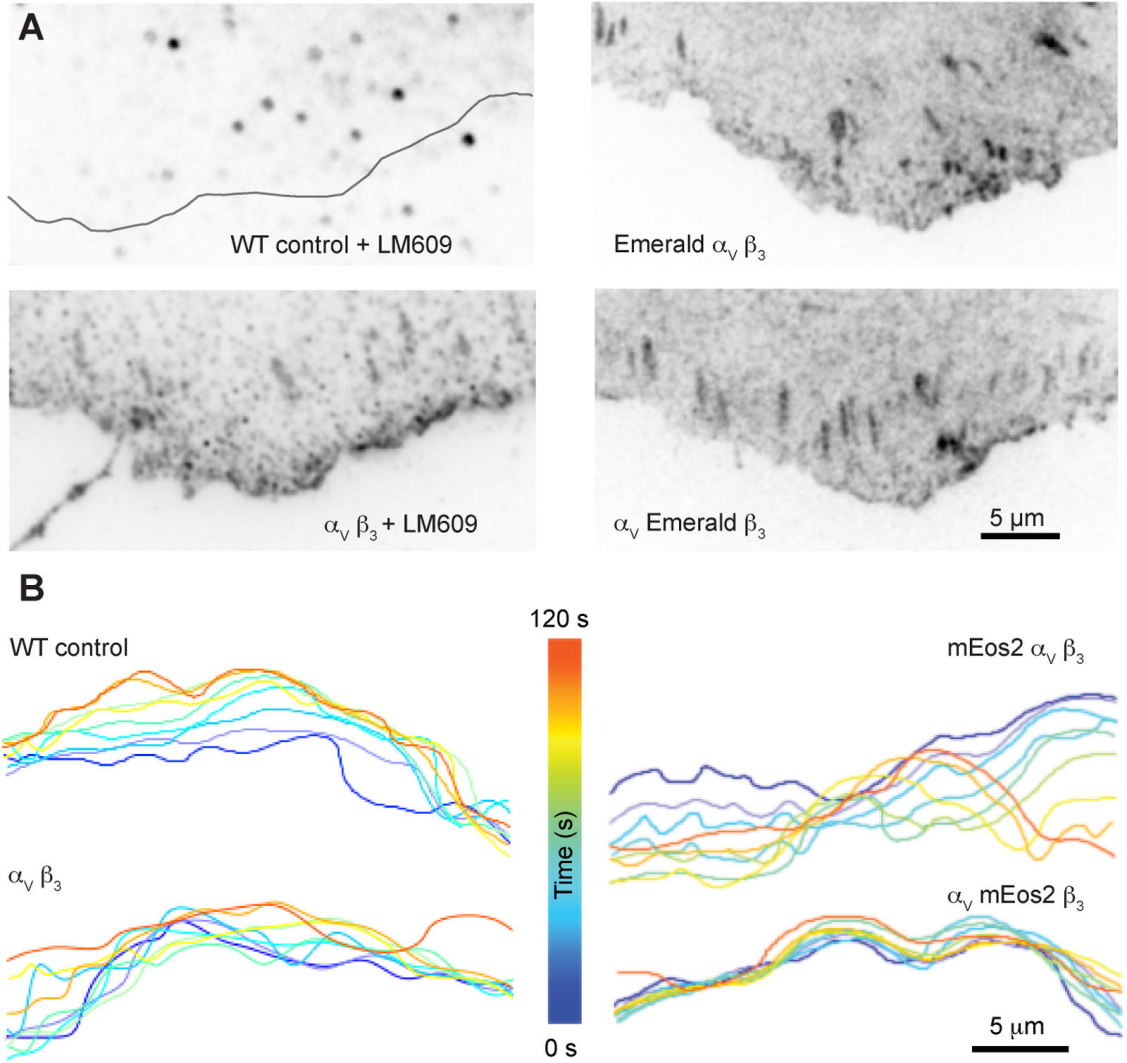
**Cellular protrusive activity slows when the beta subunit is labeled.** (A) Wild-type (WT) CHOK1 cells do not express alpha V beta 3 integrins, but co-transfected alpha V beta 3 subunits with both subunits unlabeled, Emerald labeled alpha subunits, and Emerald labeled beta subunits all localize to adhesion complexes. Unlabeled integrins are detected by the LM609 antibody. (B) Representative cell edge contours plotted every 15 s and color-coded for time show less cell protrusive activity when cells express integrins with labeled beta subunits.

We then sought to determine if these decreases in integrin mobility effect anchorage-dependent behavior by recording the protrusive activity of the cell edge. We replaced the mEos2 labels with mEmerald, and 24 h post-transfection collected time-lapse images of cells plated on fibronectin. We again used quantitative fluorescence of a co-transfected null vector to select cells with comparable integrin expression levels. Since we were imaging the cell, not the integrins, we included untransfected cells and cells transfected with unlabeled integrins in these experiments to establish native behaviors. While control experiments verified that the transfected integrins localize to adhesion complexes independently of how they were labeled (Fig. 2A), how the molecules were labeled had an effect on leading edge activity. Untransfected cells, cells transfected with unlabeled integrins and cells transfected with alpha labeled integrin subunits all behaved similarly (Fig. 2B), but the leading edges of cells transfected with labeled beta subunits were consistently more quiescent (Fig. 2B). This quiescence of the leading edge in the cells transfected with the labeled beta subunit suggests that labeling the beta subunit is increasing cell-ECM binding.

### Adhesion size varies with subunit labeling

**Fig. 3.**
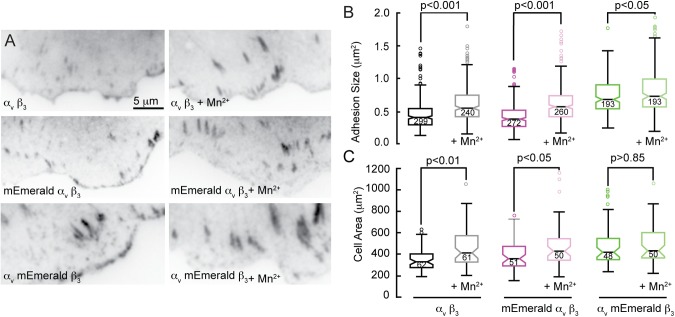
**Mn^2+^ treatment and labeling the beta subunit increase adhesion size and cell area.** (A) Micrographs of focal adhesion identified by paxillin in CHOK1 cells transfected with indicated integrins suggest an increase in size with Mn^2+^ stimulation and when beta subunits are labeled. (B) Analysis of adhesion size and in cells expressing unlabeled, alpha labeled, and beta labeled integrins. Number of adhesions analyzed indicated on box and whiskers. Number of cells analyzed per category from left to right: 33, 30, 21, 23, 33, and 34. (C) CHOK1 expressing unlabeled and alpha labeled subunits increase spreading with Mn^2+^ treatment, but cells expressing beta labeled subunits do not. Number of cells analyzed indicated on box and whiskers. *P* values are derived from two-tailed *t*-test. ANOVA and Scheffé multiple comparisons across all conditions in B and C indicate that adhesions with unlabeled and alpha labeled integrins are not statistically different (*P*<0.01).

Since increased affinity for ECM can be manifested at the cellular level by larger adhesion complexes (Cluzel et al., 2005), we next investigated whether labeling the beta subunit had an effect on the size of adhesion complexes. CHO-K1 cells were transfected with tdTomato paxillin as well as unlabeled, alpha labeled, and beta labeled integrins. Although paxillin and integrin labels identified the same adhesions (Fig. S1), measuring adhesion size with paxillin eliminated any potential bias in size between genetic expression and immunofluorescence, and allowed the paxillin intensity to serve as a metric for the expression level of co-transfected molecules (Shroff et al., 2008, 2007). All cells were plated on fibronectin-coated coverslips for 4 h, and a subset of cells from each transfection were treated during the final hour of incubation with 0.5 mM Mn^2+^ to chemically force integrin activation (Ballestrem et al., 2001). We discovered that while adhesion size increased with Mn^2+^ treatment, there were no statistical differences between adhesions formed with unlabeled or alpha labeled subunits (Fig. 3A,B). In contrast, adhesions formed with labeled beta subunits were significantly larger. Moreover, unlike the unlabeled or the alpha labeled adhesions, the beta labeled adhesions did not increase in size in response to Mn^2+^ (Fig. 3B). These results were not due to co-transfection with paxillin, since control cells exhibited the same integrin labeling-dependent adhesion size relationship (Fig. S2). Thus, the similarity in size between the unlabeled and alpha labeled adhesions as well as between the beta labeled and Mn^2+^ treated adhesions further support the interpretation that labeling the beta subunit is activating the integrin.

### Conditions that slow integrin mobility increase whole cell response to ECM

Since the slower integrin mobility with labeling was measured for integrins outside of adhesion complexes, we wanted to connect this difference in molecular behavior to how the cell surface interacted with ECM by measuring cell spreading. Cells were again transfected with paxillin, unlabeled, alpha labeled and beta labeled integrin subunits. Approximately 24 h post transfection, cells were trypsinized and separated into two groups. One group was untreated and the other group was treated with Mn^2+^; both groups were allowed to spread for 90 min prior to fixation and imaging. Fiji was used to threshold images and measure cell areas (Schindelin et al., 2012). Control experiments indicate that the transfected integrins enhance the endogenous cell response to fibronectin (Fig. S3) (*P*<0.001). But, there was no statistical difference in spreading between cells expressing unlabeled integrins or alpha labeled subunits (Fig. 3C), and Mn^2+^ treatment increased the size of the cells in both groups to be comparable to cells expressing beta labeled subunits. Significantly, Mn^2+^ did not increase the size of cells that were transfected with beta labeled subunits (Fig. 3C). These data suggest that labeling the alpha subunit does not alter whole cell affinity for ECM, but labeling the beta subunit increases cellular-ECM affinity, similar to Mn^2+^ treatment.

### Labeling the beta subunit exposes the ligand induced binding site

To directly test whether these changes in integrin affinity correspond to changes in integrin conformation, we expressed alpha 5 and beta 1 subunits in CHOB2 cells, which are an alpha 5 deficient cell line (less than 2% endogenous expression) derived from CHOK1 cells (Schreiner et al., 1989). This allowed us to measure whether subunit labeling activates the beta 1 integrin subunit by quantifying exposure of the activation epitope that the commercially available antibody, 9EG7 detects with Mn^2+^ treatment (Bazzoni et al., 1995). 9EG7 detects primed integrins, conformationally extended and activated but not necessarily ligand bound (Galbraith et al., 2007; Su et al., 2016). We first performed control cell-spreading experiments with this cell line and integrin, and we found that although the increased spreading of cells transfected with labeled alpha 5 subunits may indicate greater surface expression compared to unlabeled subunits, the CHOB2 spreading assay results were essentially identical to those obtained with CHOK1 cells; beta subunit labeling increases cell spreading. In addition, Mn^2+^ increased the spreading of the cells transfected with unlabeled and alpha labeled integrin subunits, but Mn^2+^ did not increase the spreading of cells transfected with labeled beta subunits (Figs S3 and S4).

**Fig. 4.**
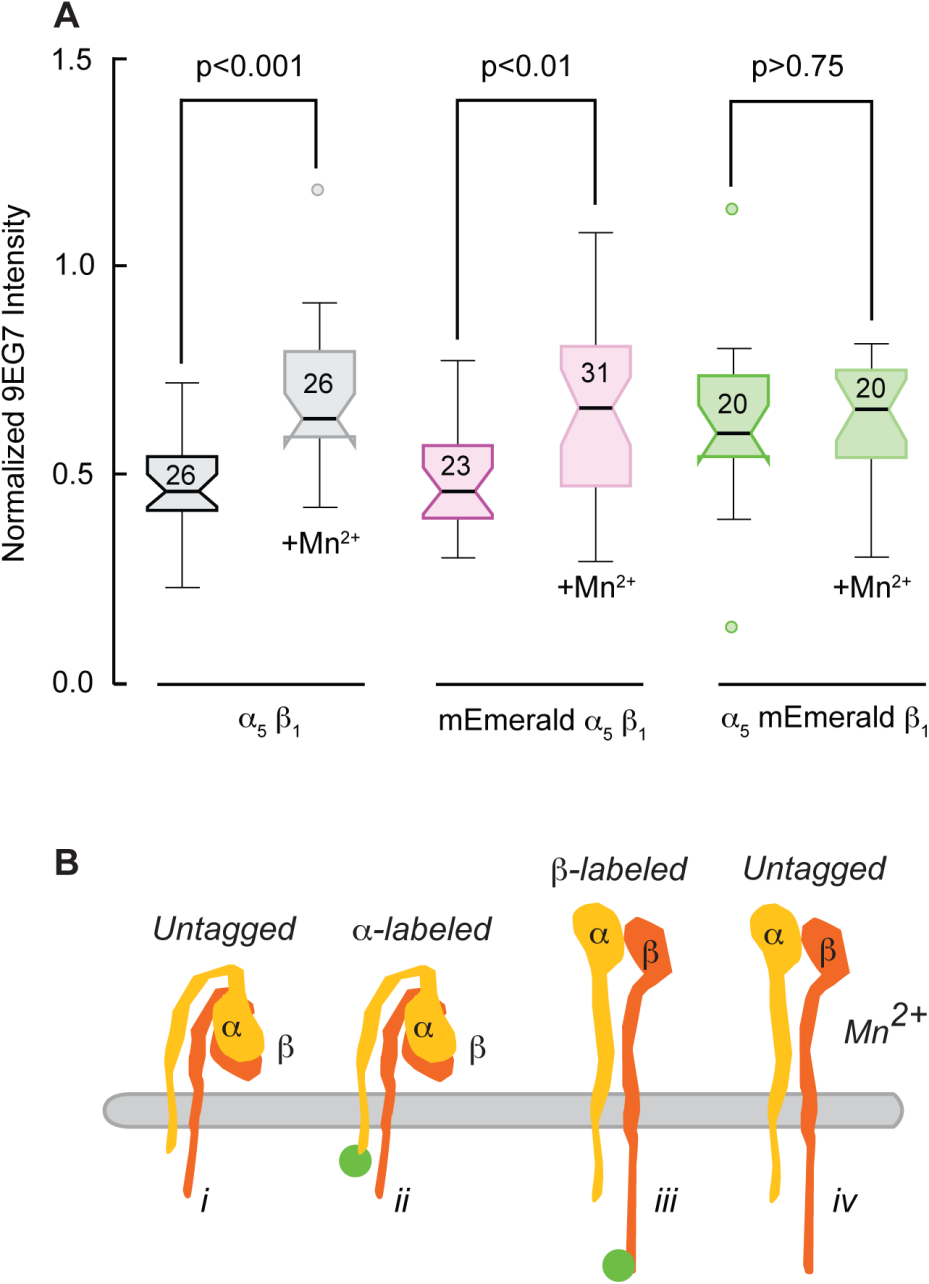
**Labeling the cytoplasmic tail of the beta subunit increases the affinity state of integrin heterodimers.** (A) Quantitative fluorescence ratio of 9EG7, an antibody that detects the extended conformation of beta 1, to K20, a non-inhibitory anti-human beta1 antibody. Intensity measured at the periphery of CHOB2 cells transfected with unlabeled human alpha 5 beta 1, alpha labeled, and beta labeled subunits. Mn^2+^ increases activation of unlabeled and alpha labeled integrins, but not beta labeled integrins. *P* values are from two-tailed *t*-tests. ANOVA and Scheffé multiple comparison indicates no significant difference between unlabeled and alpha labeled integrins for both control and Mn^2+^ treatment. Number of cells analyzed indicated on box and whiskers. (B) Cartoon illustrating the four-different integrin states: (i) unlabeled integrin, (ii) labeled alpha subunit, (iii) labeled beta subunit, and (iv) unlabeled integrins exposed to Mn^2+^.

We then measured integrin activation by quantifying the immunofluorescence intensity of 9EG7. We normalized the 9EG7 signal in a 1.5 µm wide band along the leading edge to the immunofluorescence intensity of K20, a non-function blocking anti-human beta1 antibody to account for any differences in integrin surface expression between cells. Control experiments using a secondary antibody cross-absorbed against hamster IgG confirmed that 9EG7 only labeled the cells transfected with human integrin subunits; it did not detect endogenous hamster beta 1 integrins in untransfected cells (95% positive identification by an observer blinded to the experiment). In addition, the CHOB2 cells lack alpha V and beta 3 integrins and did not have prominent adhesion complexes identified by paxillin that could have biased the fluorescence intensity measurements. We found that integrin activation increased with Mn^2+^ treatment for unlabeled integrins and integrins with alpha 5 labeled subunits (Fig. 4A). In contrast, integrins with labeled beta 1 subunits and unlabeled integrins treated with Mn^2+^ both had elevated affinity levels that were not statistically different from each other (Fig. 4A). Although some of our transfected subunits could be forming heterodimers with endogenous subunits, our data indicates that fluorescently labeling the beta subunit elevates the affinity state for multiple integrin heterodimers, similar to Mn^2+^ treatment (Fig. 4B).

Similar to our results, previous direct comparison of GFP-labeling of either the alpha IIb or the beta 3 integrin subunits reported that the integrin heterodimer properly localized and the cells retained their ability to perform anchorage-dependent functions, independently of which subunit was labeled (Plançon et al., 2001). However, it was also noted that cells expressing beta labeled subunits were more likely to spontaneously aggregate in the presence of a soluble ligand (Plançon et al., 2001). Our comparison of unlabeled, alpha labeled, and beta labeled integrins in untreated cells and in cells treated with Mn^2+^ suggests that the spontaneous aggregation of cells with labeled beta subunits is consistent with integrin activation. Additionally, our activation epitope measurements indicate that labeling the beta subunit changes the integrin from the bent inactive conformation to the extended conformation by disrupting the disulfide bond between the calf-2 and β-tail domains (Su et al., 2016). Taken together, these data suggest that evaluating whether a labeled protein reports native protein function by the ability of the population to properly localize does not account for the functional state of the protein at the molecular level.

Here, we measured the functional state of the protein and connected the significantly lower mobility of integrins with beta labeled subunits to conformational changes in the molecule and to modified cellular adhesion-dependent behaviors; less dynamic leading edges, larger adhesions, and larger surface areas. The changes in cellular behaviors were significant, but not so grossly abnormal that they would not have been attributed to biological variability unless they were connected to molecular behaviors or compared to the behaviors of cells with unlabeled proteins. Thus, our findings indicate that changes in dynamic integrin behaviors can be coupled to differences in molecular conformation as well as cellular adhesion-dependent function, demonstrating a connection between measured molecular behaviors and distinct cellular outputs.

## MATERIALS AND METHODS

### Cell culture and transfection

CHOK1 and CHOB2 cells were grown in DMEM-F12 supplemented with 10% FBS. Cells were transfected with either alpha V and beta 3 (CHOK1), or alpha 5 and beta 1 (CHOB2) using a Nucleofector II (Lonza) and Ingenio (Mirus) transfection reagents following the manufacturer's protocols. Unlabeled integrins were in either pcDNA3.1 (alpha V, beta 3 and alpha 5) or pRK5 vectors (beta 1), and labeled vectors (mEos2 or mEmerald) were constructed as previously described (Jaqaman et al., 2016). The labeled vectors are available through Addgene, with the Addgene number listed in parenthesis following the vector name. Detailed maps and sequences of the inserts and linkers are available on the Addgene website. The plasmids are: mEos2-Alpha-V-integrin-N-25 (57345), mEmerald-Alpha-V-integrin-N-25 (53985), mEos2-Integrin-Beta3-N-18 (57391), mEmerald-Beta3-N-18 (54130), mEmerald-Alpha5-Integrin-12 (53984), and mEmerald-Beta1-N-18 (54129), tdTomato Paxillin-22 (58123). The unlabeled alpha V and beta 3 subunits were gifts from Mark Ginsberg (UCSD). Cells transfected with mEos2 labeled subunits were also transfected with an EFGP vector to identify edge contours in live cell experiments. Cells were plated on plasma-etched cover glass that had been silanized and coated overnight with either 5 µg/ml human plasma fibronectin (CHOK1) or 10 µg/ml human plasma fibronectin (CHOB2).

### Cell spreading and integrin activation assays

Approximately 24 h after transfection, CHOK1 cells were trypsinized and plated for 90 min for spreading assays or 4 h for adhesion assays. Cells were then fixed with 2% paraformaldehyde in PHEM (Galbraith et al., 1998). For activation of CHOK1 cells, treatment with 0.05 mM Mn^2+^ was initiated 5 min prior to plating and was maintained throughout the spreading assay (Humphries, 2001). To visualize unlabeled alpha V beta 3 integrins, fixed cells were stained with LM609 (Millipore) and an Alexa 488 secondary antibody. To quantify the amount of integrin activation transfected CHOB2 cells were plated overnight prior to fixation and subsequently labeled with 9EG7 (BD Pharmigen, San Diego, USA) and a CY5 highly cross-absorbed secondary antibody (Jackson ImmunoResearch), with cross-absorption species including mouse, rat, guinea pig and hamster. 9EG7 interacts with high affinity mouse and human beta 1 integrin (Galbraith et al., 2007), binding the ligand-induced binding (LIBS) epitope exposed by Mn^2+^ treatment (Bazzoni et al., 1995). Activation of CHOB2 with 0.05 mM Mn^2+^ was initiated 1 h prior to fixation. These cells were also labeled with K20 (Beckman Coulter, Brea, USA), a non-inhibitory monoclonal anti-beta 1 antibody that also reacts with human integrin. K20 was labeled with an Alexa 565 secondary antibody. Control experiments confirm that 9EG7 does not interact with endogenous hamster integrin.

### Microscopy

All imaging experiments were performed on an Olympus IX71 with a 60×1.49 NA objective using TIRF illumination. To create the TIRF beam, four laser lines (405, 488, 561, 633 nm) (Coherent) were merged and introduced through free space into the TIRF illumination port of the microscope as previously described (Jaqaman et al., 2016). Position of the beam in the back aperture of the objective was motorized to ensure repeatability of the penetration depth of the evanescent TIRF wave. Thus, fluorescence intensity could be compared between cells and between experiment repetitions because the excitation power and the depth were all digitally controlled.

For the single-molecule experiments, mEos2 labeled molecules were imaged with a low level of 405 nm activation and 561 nm excitation light (5 µW and 2.5 mW at the back aperture, respectively). In the single-molecule experiments, every 10 s (400 frames) the excitation light was switched to 488 nm (100 µW at the back aperture) using an acousto-optic tunable filter (AOTF, AA Opto-Electronic, Orsay, France) to image the unconjugated EGFP filling the cell. Experiments were imaged at 37°C for a minimum of 5 min, and cells did not display any abnormal morphology or decreased protrusive activity that would be indicative of photodamage at the end of this interval (Shroff et al., 2008). Images were acquired at a final magnification of 111 nm/pixel with an Andor 897 EMCCD camera using an exposure time of 25 ms. To quantify activation and expression, the laser powers and TIRF settings were maintained at constant levels for all experiment replicates.

### Image processing, single-molecule analysis, and statistics

Conventional diffraction-limited micrographs were processed in Photoshop to linearly stretch contrast; Gaussian filtered with a sigma of 0.8 pixels, and then Unsharp Mask filtered with a sigma of 0.9 pixels and 75%. The cell edge and adhesions were detected by thresholding images in Fiji after smoothing with a 1 pixel Gaussian kernel sigma to reduce noise. Some adhesions were manually traced. Only fluorescence aggregates larger than 10 pixels were identified as adhesion complexes, and these adhesion complexes had an average elliptical aspect ratio of between 3.5–4.6. Canny edge detection was used on the whole cell images after thresholding to obtain cell contours.

Single-molecule analysis was performed using uTrack software (Jaqaman et al., 2008) to localize and track individual mEos2 integrin molecules. Only molecules localized to better than 25 nm precision were used for mobility analysis. Diffusion coefficients for tracks greater than 20 frames were analyzed as previously described and classified as either confined, freely diffusing or undergoing directed movement (i.e. drift) (Ewers et al., 2005; Jaqaman et al., 2016). An average of between 6000 and 7000 molecules per cell were analyzed with a minimum of 11 cells per experimental group.

One-way ANOVA analysis was performed on all experimental groups to ensure that the three to four independent experiments could be grouped for analysis. One-way ANOVA followed by Scheffé post-hoc multiple comparison test was used to identify whether any of the six treatments (unlabeled, alpha labeled, beta labeled, with and without Mn^2+^) significantly differed from each other. Untreated and Mn^2+^ treated cells with the same type of transfection were compared by two-tail *t*-tests, and these *P* values are reported in the figures.

## Supplementary Material

Supplementary information

First Person interview
