# Supplementary material for: Coupling integrin dynamics to cellular adhesion behaviors
Source: Biol Open. 2018 Aug 15;7(8):bio036806. doi: 10.1242/bio.036806 (PMC6124568; doi:10.1242/bio.036806)
Supplement: Supplementary information [file biolopen-7-036806-s1.pdf]

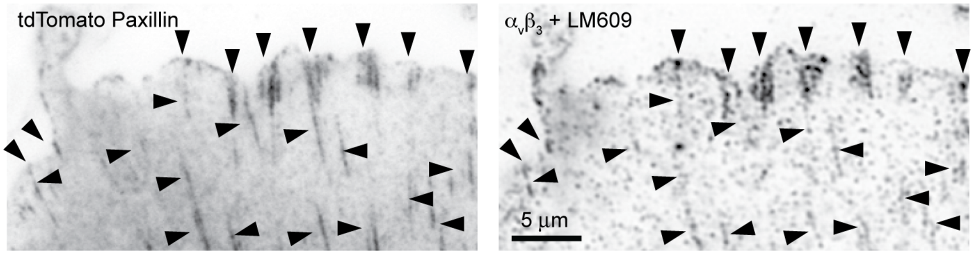


**Figure S1: Comparison of Adhesions Identified By Paxillin and Integrin Clusters.**

Transfection of tdTomato Paxillin and unlabeled alpha V beta3 stained with LM609 both identify the same focal adhesions.

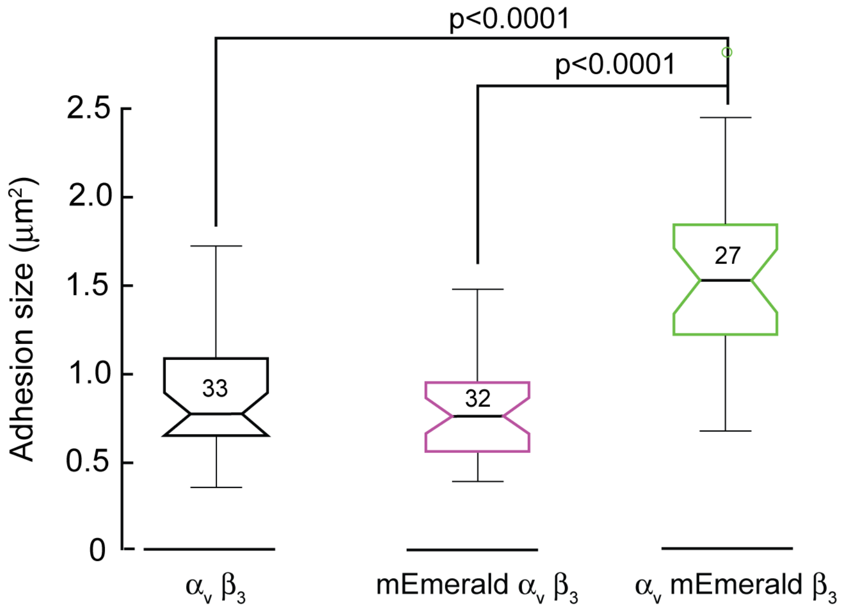


**Figure S2: Labeling the Beta Integrin Subunit Increases Adhesion Size.** Cells transfected with unlabeled, alpha labeled, and beta labeled subunits, but not co-transfected with paxillin, demonstrate that adhesions formed from unlabeled integrins are not significantly different from those formed with alpha labeled subunits, but both are significantly different from adhesions formed with beta labeled subunits. These adhesions were measured by size of mEmerald labeled integrin or LM609 stained integrin, and the data yields the same conclusion as the adhesion sizes measured with paxillin in Figure 3. P values are from ANOVA and Scheffé comparison. Numbers inside box and whiskers plots indicate n measured.

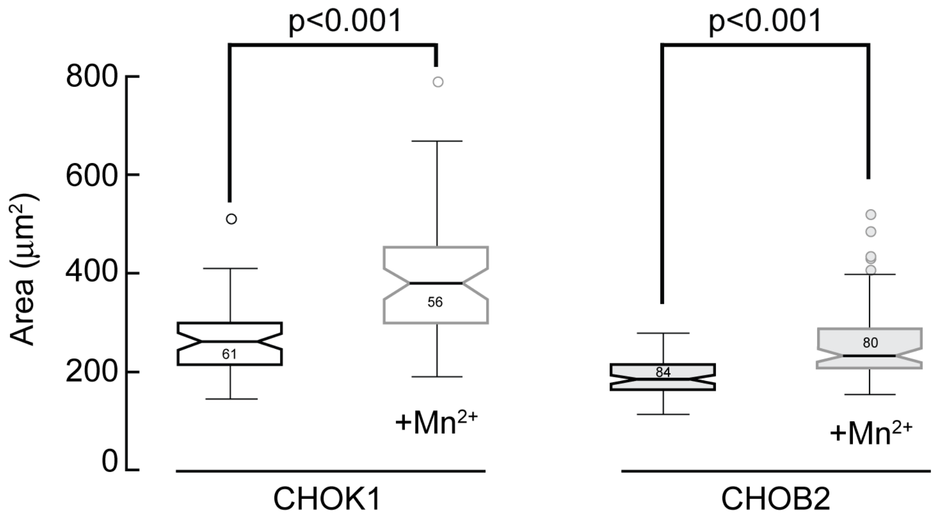


**Figure S3: Untransfected CHOK1 and CHOB2 Cells Both Increase Spreading in Response to $Mn^{2+}$.** Spreading of untransfected CHOK1 and CHOB2 cells both significantly increase in response to $Mn^{2+}$. P values are derived from two-tailed t-tests, and numbers inside box and whiskers plots indicate n measured.

Biology Open • Supplementary information

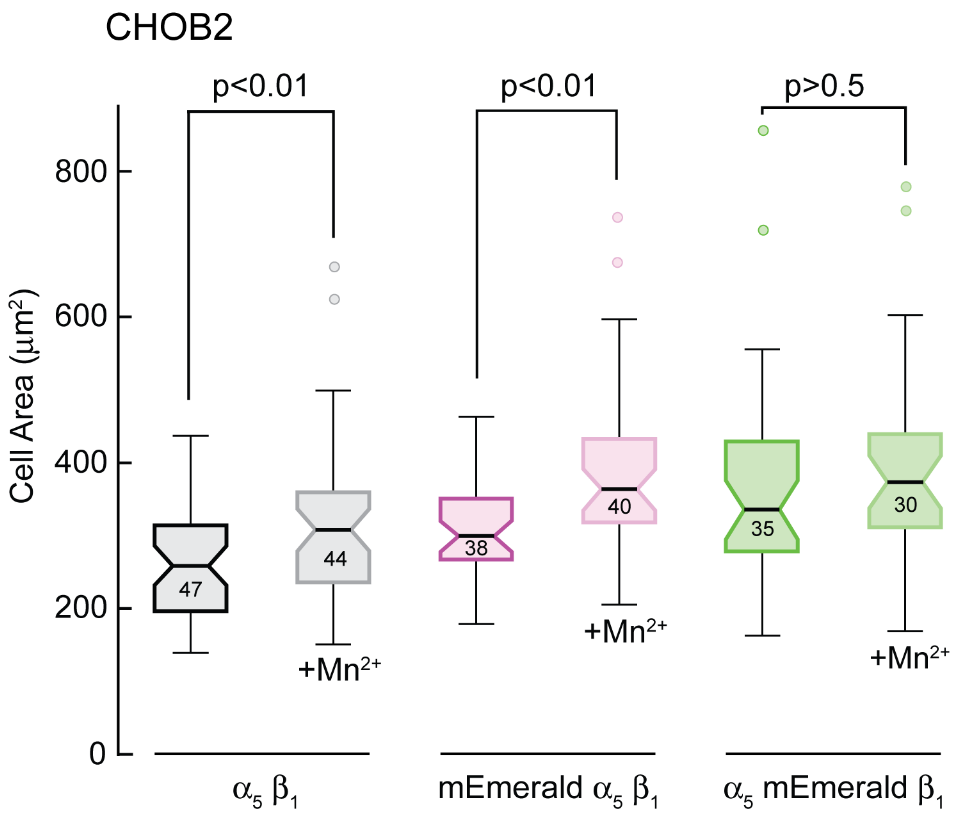


**Figure S4: $Mn^{2+}$ Treatment and Labeling the Beta Subunit Increase CHOB2 Cell Area.** CHOB2 cells transfected with unlabeled and alpha labeled alpha 5 beta 1 integrins also increase spreading with $Mn^{2+}$ treatment. CHOB2 cells, like CHOK1, do not increase in size following $Mn^{2+}$ when the cells are expressing integrins with beta labeled subunits. P values are from two-tailed t-tests between control and $Mn^{2+}$ treatments. Numbers within box and whiskers indicate the number of cells analyzed per condition.

Biology Open • Supplementary information

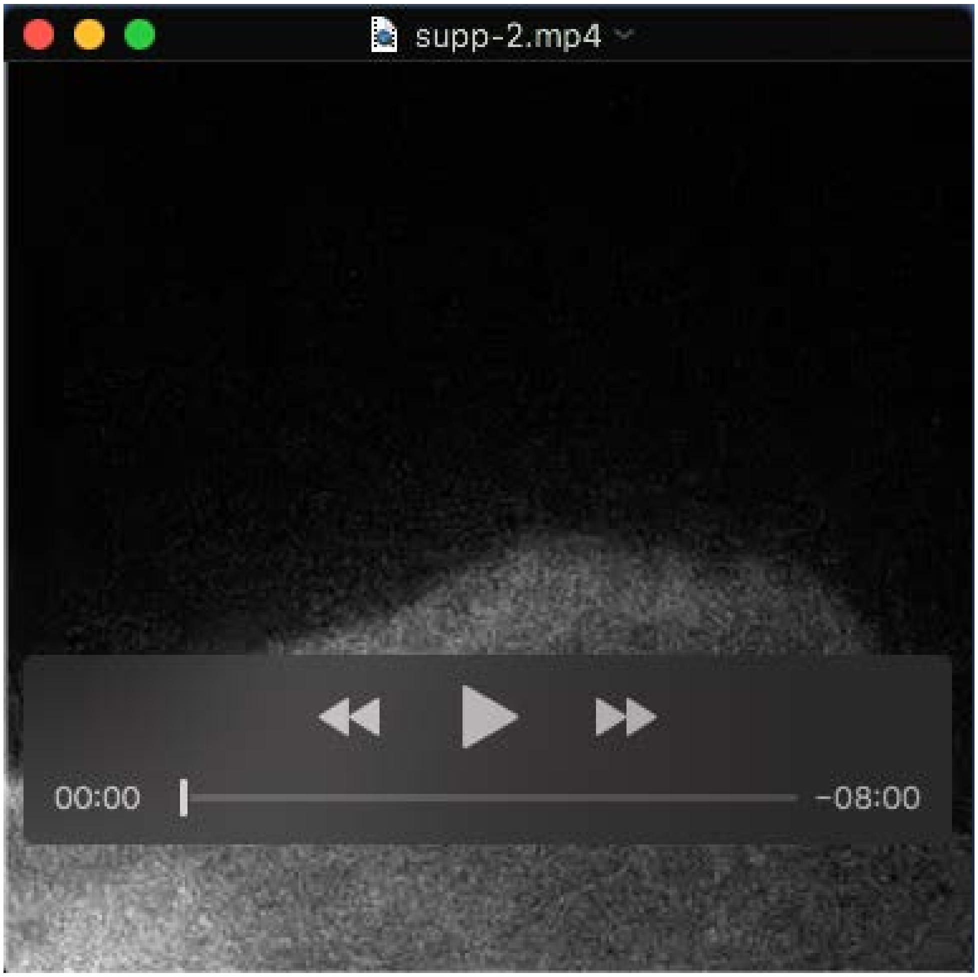


**Movie S1:** Live cell single molecule video of CHOK1 cell transfected with mEos2 $\alpha_v\beta_3$ imaged at 25 ms per frame for 2 min (4800 frames). Red circles indicate detected molecules. Interspersed every 10s (400 frames) is an EGFP fill which allows determination of the cell outline. Field size – 28.4μm x 28.4μm.
